# Characterizing the morbid genome of ciliopathies

**DOI:** 10.1186/s13059-016-1099-5

**Published:** 2016-11-28

**Authors:** Ranad Shaheen, Katarzyna Szymanska, Basudha Basu, Nisha Patel, Nour Ewida, Eissa Faqeih, Amal Al Hashem, Nada Derar, Hadeel Alsharif, Mohammed A. Aldahmesh, Anas M. Alazami, Mais Hashem, Niema Ibrahim, Firdous M. Abdulwahab, Rawda Sonbul, Hisham Alkuraya, Maha Alnemer, Saeed Al Tala, Muneera Al-Husain, Heba Morsy, Mohammed Zain Seidahmed, Neama Meriki, Mohammed Al-Owain, Saad AlShahwan, Brahim Tabarki, Mustafa A. Salih, Ciliopathy WorkingGroup, Tariq Faquih, Mohamed El-Kalioby, Marius Ueffing, Karsten Boldt, Clare V. Logan, David A. Parry, Nada Al Tassan, Dorota Monies, Andre Megarbane, Mohamed Abouelhoda, Anason Halees, Colin A. Johnson, Fowzan S. Alkuraya

**Affiliations:** 1 Department of Genetics, King Faisal Specialist Hospital and Research Center, Riyadh, Saudi Arabia; 2 Leeds Institute of Biomedical & Clinical Sciences, University of Leeds, Leeds, LS9 7TF UK; 3 Department of Pediatrics, Division of Medical Genetics, Stanford University, Stanford, CA USA; 4 Department of Pediatric Subspecialties, Children’s Hospital, King Fahad Medical City, Riyadh, Saudi Arabia; 5 Department of Pediatrics, Prince Sultan Military Medical City, Riyadh, Saudi Arabia; 6 Department of Pediatrics, Qatif Central Hospital, Qatif, Saudi Arabia; 7 Department of Ophthalmology, Specialized Medical Center Hospital, Riyadh, Saudi Arabia; 8 Department of Obstetrics and Gynecology, King Faisal Specialist Hospital and Research Center, Riyadh, Saudi Arabia; 9 Department of Pediatric, Genetic Unit, Armed Forces Hospital Southern Region, Khamis Mushayt, Saudi Arabia; 10 Department of Pediatrics, King Khalid University Hospital and College of Medicine, King Saud University, Riyadh, Saudi Arabia; 11 Human Genetics Department, Medical Research Institute, Alexandria University, Alexandria, Egypt; 12 Department of Pediatrics, Security Forces Hospital, Riyadh, Saudi Arabia; 13 Department of Obstetrics and Gynecology, King Khalid University Hospital and College of Medicine, King Saud University, Riyadh, Saudi Arabia; 14 Department of Medical Genetics, King Faisal Specialist Hospital and Research Center, Riyadh, Saudi Arabia; 15 Saudi Human Genome Project, King Abdulaziz City for Science and Technology, Riyadh, Saudi Arabia; 16 Division of Experimental Ophthalmology and Medical Bioanalytics, Center for Ophthalmology, Eberhard-Karls University Tübingen, 72076 Tübingen, Germany; 17 Institut Jerome Lejeune, Paris, France; 18 Health Information Technology Affairs, King Faisal Specialist Hospital and Research Center, Riyadh, Saudi Arabia; 19 Department of Anatomy and Cell Biology, College of Medicine, Alfaisal University, Riyadh, Saudi Arabia

**Keywords:** Cilia, Bardet-Biedl, Joubert, Meckel-Gruber, Nephronophthisis, Acrocallosal, Senior-Loken, Polycystic kidney, Oral-facial-digital, Founder, Variability, Modifier, Oligogenic

## Abstract

**Background:**

Ciliopathies are clinically diverse disorders of the primary cilium. Remarkable progress has been made in understanding the molecular basis of these genetically heterogeneous conditions; however, our knowledge of their morbid genome, pleiotropy, and variable expressivity remains incomplete.

**Results:**

We applied genomic approaches on a large patient cohort of 371 affected individuals from 265 families, with phenotypes that span the entire ciliopathy spectrum. Likely causal mutations in previously described ciliopathy genes were identified in 85% (225/265) of the families, adding 32 novel alleles. Consistent with a fully penetrant model for these genes, we found no significant difference in their “mutation load” beyond the causal variants between our ciliopathy cohort and a control non-ciliopathy cohort. Genomic analysis of our cohort further identified mutations in a novel morbid gene *TXNDC15*, encoding a thiol isomerase, based on independent loss of function mutations in individuals with a consistent ciliopathy phenotype (Meckel-Gruber syndrome) and a functional effect of its deficiency on ciliary signaling. Our study also highlighted seven novel candidate genes (*TRAPPC3*, *EXOC3L2*, *FAM98C*, *C17orf61*, *LRRCC1*, *NEK4*, and *CELSR2*) some of which have established links to ciliogenesis. Finally, we show that the morbid genome of ciliopathies encompasses many founder mutations, the combined carrier frequency of which accounts for a high disease burden in the study population.

**Conclusions:**

Our study increases our understanding of the morbid genome of ciliopathies. We also provide the strongest evidence, to date, in support of the classical Mendelian inheritance of Bardet-Biedl syndrome and other ciliopathies.

**Electronic supplementary material:**

The online version of this article (doi:10.1186/s13059-016-1099-5) contains supplementary material, which is available to authorized users.

## Background

The primary cilium is a microtubule-based projection in non-dividing cells which serves as a sensory organelle and signaling hub that mediates numerous physiological roles [1, 2]. Originally viewed as a cellular appendage of unclear significance, defects of the cilium are now known to cause clinically recognizable developmental syndromes (ciliopathies), the phenotypic spectrum of which involves nearly every body organ [3, 4]. Although ciliopathies are conveniently classified into specific syndromes, their phenotypes are best viewed as a continuum that spans a phenotypic spectrum from embryonic lethality to isolated late onset retinal degeneration [5]. Several studies support this view by demonstrating that individual ciliopathy disease genes are expressed broadly rather than discretely across the spectrum, and that mutations within the same gene can display marked phenotypic differences across and even within families [6, 7]. The mechanisms underlying these variations in expressivity are unknown but likely involve modifier alleles as well as stochastic events [8–10].

Annotating the morbid genome of ciliopathies can greatly expand our knowledge about the non-redundant components of the “ciliome,” and the annotation has progressed dramatically with the advent of massive parallel sequencing [11]. However, the annotation of the morbid genome remains incomplete, and the suggestion that its missing part lies in non-Mendelian forms of inheritance remains controversial [12]. We have recently shown that the hypothetical yield of exome sequencing in the setting of autosomal recessive diseases is >95% and that this can be achieved with the aid of positional mapping where applicable [13]. The study of ciliopathies in an inbred population, therefore, presents an opportunity to not only annotate the morbid genome of ciliopathies more fully but also test the possible contribution of non-Mendelian inheritance to these conditions.

## Results

### Defining the ciliopathy phenotypic spectrum

**Fig. 1:**
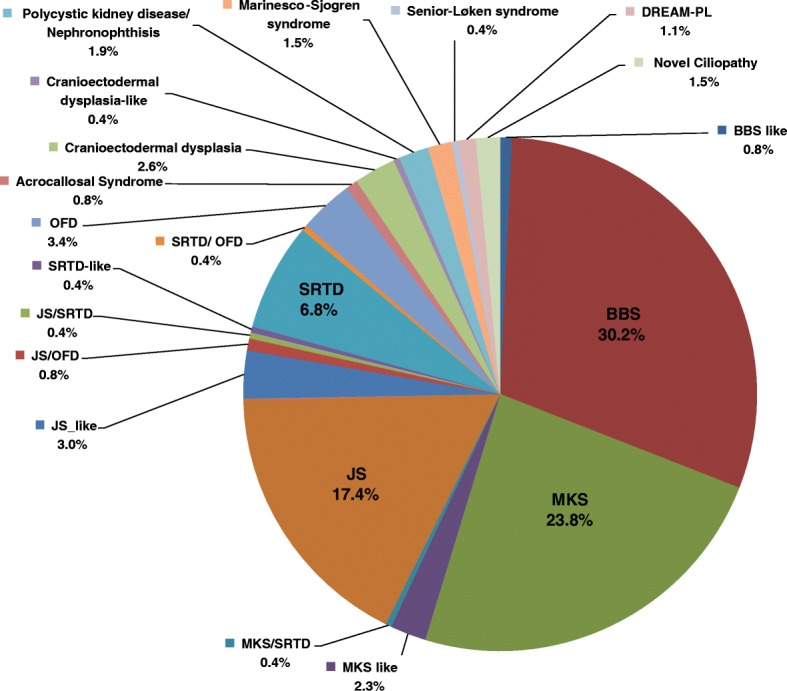
A pie chart representation of the distribution of the different ciliopathy syndromes included in the study cohort

Although we have actively sought and accepted referral of cases with phenotypes that fall anywhere along the ciliopathy spectrum, the contribution of individual syndrome categories varied greatly among the 265 families recruited in this study; e.g., Bardet-Biedl syndrome alone accounted for 31% (Fig. 1). The large size of our cohort allowed us to calculate the frequency of the main features for each distinct ciliopathy syndrome (see Additional file 1: Figure S1). Consistent with ciliopathies being a spectrum of phenotypes, we show that 9% (23/265) of families did not conform fully to a specific ciliopathy syndrome and were labeled as similar to the closest matching syndrome. However, seven families appeared to have recognizable syndromic presentations that are distinct from all known ciliopathy syndromes (Additional file 2: Table S3). The clinical features of the index (15DG2466) for one of these families with mutation in *NEK4* are described in Additional file 3: supplemental clinical data. In three families, the affected members were found to share similar phenotypes, which have been recently published with the acronym DREAM-PL to highlight the main clinical features (dysmorphic facies, renal agenesis, ambiguous genitalia, microcephaly, polydactyly, and lissencephaly) [14, 15]. The fifth family displayed congenital hydrocephalus, synpolydactyly, and complex congenital heart disease, and no likely candidate was identified on exome sequencing. The clinical phenotype of the affected member in family 6 comprised severe microcephalic primordial dwarfism, lissencephaly, ambiguous genitalia, and anophthalmia. Family 7 displayed polydactyly and abnormal gyration characterizing malformation of cortical development. No candidate causal variant was identified in family 6 or 7.

### The ciliopathy morbid genome

A likely causal variant was identified in 85% (225/265) of the studied families, spanning 54 known genes that have been previously linked to ciliopathy phenotypes (see Additional file 2: Table S3). All affected individuals were confirmed to carry biallelic variants in these genes except for hemizygous mutations in *OFD1*. Of note, several of these variants had evaded clinical sequencing, usually because they were intronic, and were subsequently identified through our autozygome-guided RT-PCR analysis of the target genes (Additional file 4: Table S2). In family 81, three BBS members were negative on clinical exome, but through homozygosity mapping and RT-PCR we identified the absence of exons 13–17 of *BBS1* only at the complementary DNA (cDNA) level. The splice site mutation in *SIL1* (NM_022464.4: c.1030-9G > A: p. (Phe345Alafs*9)) was also missed by clinical exome in family 254, and through homozygosity mapping and RT-PCR we were able to confirm the pathogenicity of this mutation. Family 18 represents a third example of a family who had a negative result from the clinical exome but was subsequently found to have a genomic deletion (exons 8–15) in *BBS2* (NM_031885.3: c.805_1910del: p. (Val269Glufs*12)) through homozygosity mapping and direct Sanger sequencing of *BBS2*. We estimate that conventional exome sequencing could have missed 10% of the likely causal variants identified in our study based on the contribution of non-canonical splicing mutations. This may explain why previous studies suggested lower estimates for the total contribution of known disease genes to the etiology of ciliopathies.

**Fig. 2:**
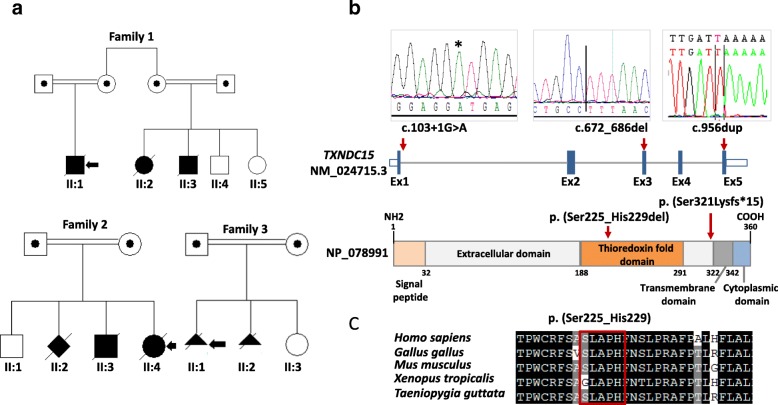
*TXNDC15* is a novel gene that causes Meckel-Gruber syndrome. **a** Pedigree of the three families showing the consanguineous nature of the parents. The index is indicated in each pedigree by a *black arrow*. **b** *Upper panel*: sequence chromatogram for the three homozygous mutant alleles in *TXNDC15* and their locations indicated in a schematic of *TXNDC15. Lower panel*: schematic of the TXNDC15 protein and the location of the mutations in each specific domain. **c** Multisequence alignment of the deleted five amino acid residues (p. (Ser225_His229del)) showing high conservation of this part of amino acids down to *Taeniopygia guttata* (*boxed in red*)

**Fig. 3:**
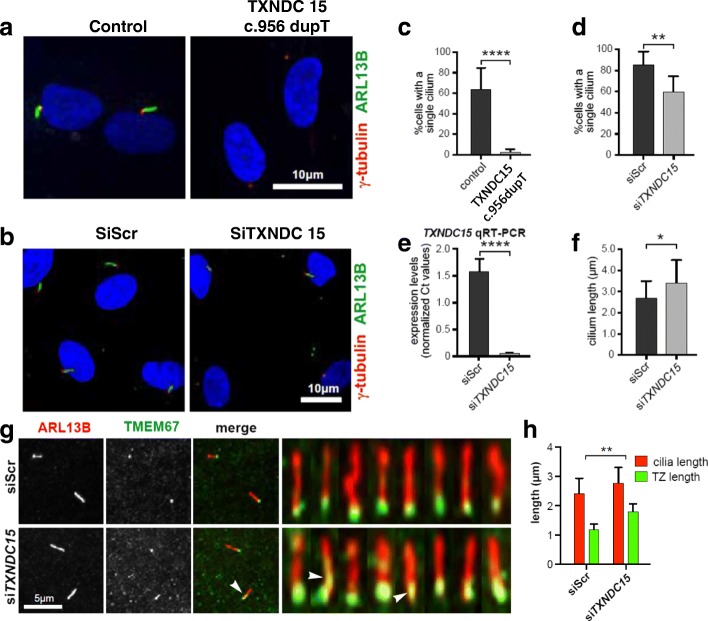
Mutation in *TXNDC15* causes ciliogenesis defect in patient cells and aberrant localization of TMEM67 using *TXNDC15* RNAi. **a** and **b** Immunofluorescence images of serum-starved fibroblasts from the affected individual in family 3 (II:1) and control fibroblasts (**a**) and *TXNDC15* siRNA in human hTERT-RPE1 cells (**b**) stained for the ciliary marker ARL13B (*green*), gamma tubulin (*red*), and DNA (*blue*). Compared to controls, fibroblasts from II:1 in family 3 and *TXNDC15* siRNA knockdown showed a marked ciliogenesis defect. **c** Bar graph showing the significant reduction in the number of ciliated fibroblast cells derived from individual II:1 from family 3. **d** Bar graph showing a significant reduction in the number of ciliated *TXNDC15* siRNA cells compared to negative control scrambled siRNA. **e** Bar graph showing the efficiency of *TXNDC15* siRNA compared to negative control scrambled siRNA (*siScr*) as quantified by qRT-PCR for the *TXNDC15* transcript. **f** Bar graph showing the average increase in the cilium length following *TXNDC15* siRNA knockdown compared to negative control scrambled siRNA (*siScr*). **g** Immunofluorescence microscopy images of serum-starved hTERT-RPE1 cells following *TXNDC15* siRNA knockdown stained for the ciliary marker ARL13B (*red*), the transition zone marker TMEM67 (*green*), and DNA (*blue*) showing the incorrect localization of the TMEM67 ciliary receptor to the transition zone compared to negative control scrambled siRNA (*siScr*). **h** Bar graph showing an elongation in the total cilia length and elongation of the transition zone due to mislocalization of TMEM67 (*arrowheads* in **g**). Statistical significance of pair-wise comparisons are indicated by * *p* < 0.05, ** *p* < 0.01, and **** *p* < 0.0001 (panels **c**–**f**, Student’s paired t test for *n* = 3 biological replicates; and panel **h**, Pearson’s chi-squared test for *n* = 3 biological replicates)

Of the remaining 40 families, nine (22.5%) had variants in eight novel candidate ciliopathy genes (*TXNDC15, TRAPPC3*, *EXOC3L2, FAM98C, C17orf61*, *LRRCC1*, *NEK4*, and *CELSR2*). One of these candidate genes, *TXNDC15*, was independently mutated in two families that share the cardinal features of Meckel-Gruber syndrome (see Additional file 3: supplemental clinical data). Furthermore, through an international collaboration, we were able to identify an additional Meckel-Gruber syndrome patient with a homozygous truncating variant in this gene (Fig. 2 and Additional file 3: supplemental clinical data). Although *TXNDC15* did not localize to the primary cilium or periciliary regions (data not shown), patient fibroblasts (Fig. 3a) as well as cells subjected to siRNA knockdown (Fig. 3b) had aberrant ciliogenesis (Fig. 3c–f). *TXNDC15* encodes a putative protein disulfide isomerase that contains a thioredoxin domain. Proteomic studies showed that TXNDC15 interacted with a total of 224 endomembrane-associated proteins after filtering (Additional file 5: Table S4) that were significantly enriched in known or predicted ciliary proteins (SYSCILIA Gold Standard, SCGSv1 [16]; *p* = 2.34 × 10^-18^ hypergeometric test, observed 39, expected 7.02). Furthermore, loss of TXNDC15 prevented correct localization of the TMEM67 ciliary receptor to the transition zone (Fig. 3g and h). Taken together, our data support a causal role for the biallelic truncating mutations we identified in *TXNDC15* and the Meckel-Gruber syndrome phenotype in these patients.

### Ciliopathies are Mendelian disorders

The high concordance between the percentage of ciliopathy patients with likely causal variants (85%, excluding novel candidates) and the hypothetical maximum contribution of genic (in contrast to mutations in non-genic DNA) mutations to autosomal recessive diseases (95%, see above Shamseldin et al. 2016, *Genetics in Medicine*, in press) supports the view that ciliopathies are indeed Mendelian disorders. To further test this, we asked whether the burden of rare (MAF <0.01) variants in known ciliopathy genes (total 89 genes) in our cohort is higher than that expected by chance. To this end, we calculated the burden of these alleles in all 31 individuals in our cohort with exome sequencing data and in a “control” cohort of 64 intellectually disabled patients from which ciliopathy cases have been specifically excluded, and found no statistically significant difference between the two cohorts (average numbers of variants per sample 7.0 and 6.98, respectively; two-tailed unpaired Student’s *t* test, *p* = 0.91122). Not all rare alleles are pathogenic, and it is possible that patients with ciliopathies are specifically enriched in pathogenic rare alleles, as previously suggested. In order to test this, we repeated our analysis and only scored for alleles with MAF <0.01 and CADD >20. Again, there was no significant difference between the two cohorts (1.129 vs. 1.187 (*t* test: *p* = 0.68218)).

### Phenotype/genotype correlation in ciliopathies

**Fig. 4:**
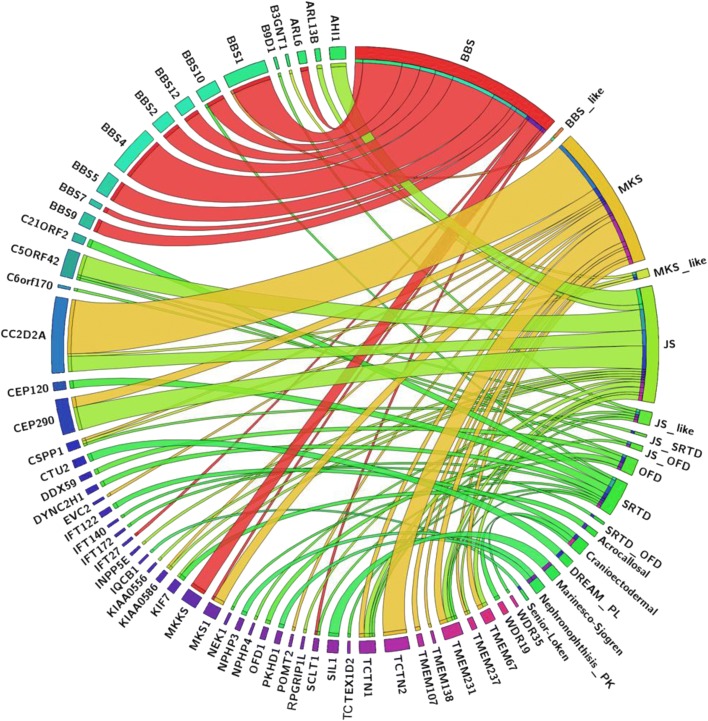
Circos image showing the different ciliopathy disorders in relation to known ciliopathy genes

**Fig. 5:**
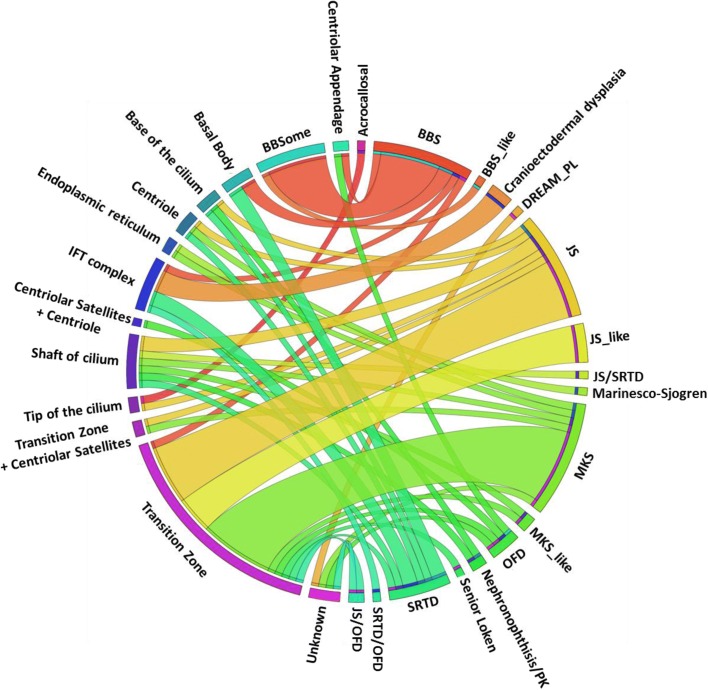
Circos image showing the different ciliopathy disorders in relation to ciliary compartment

The spectrum of phenotypes observed for a given ciliopathy gene ranged from broad, e.g., four distinct syndromes were observed in the context of mutations in *TCTN1*, to very narrow, e.g., all 16 mutations in *BBS1* caused one single phenotype (Fig. 4). Two different mutations in *TCTN1* (NM_001082538.2:c.32_43del: p. (Val11_Leu14del) and NM_001082538.2:c.1385dupT: p. (Trp463Valfs*58)) were identified in patients with MKS, while a splice site mutation (NM_001082538.2:c.342-2A > G) was identified in two families, one with JS_like, and a fourth family with JS/OFD. However, we also observed a trend where genes that cluster in the same ciliary compartment tended to cause a similar phenotype; e.g., mutations in the transition zone genes *CC2D2A*, *TCTN2*, *TMEM237*, and *CEP290* almost always resulted in either Joubert or Meckel-Gruber syndrome phenotypes (Fig. 5).

One advantage of our study cohort was the opportunity to observe the phenotypic expression of individual alleles not only among several siblings due to large family size, but also across many families due to the tribal structure and founder effect in the study population [17]. Thus, one can test the relative contributions of either modifier alleles or stochastic effects by comparing the degree of phenotypic consistency among siblings (implying a minimal variation of modifier alleles) with unrelated individuals who share the same founder allele (maximum variation of modifier alleles). In families with available clinical data on the affected siblings of the index, 97% (69/71 families) belonged to the same disease category, compared to 92% (34/37) of unrelated patients with ciliopathies who shared the same founder mutation. Another approach for looking at interfamilial variability is to calculate the concordance in the disease categories between patients we report in this study and those previously reported with the same mutation. Again, for 78 such variants, we observed a very high concordance in the disease category (76/78, 97%). Interestingly, the only two variants for which we observed discordance between the reported phenotypes and the ones we observe in our cohort were in the compound heterozygous state with other mutations (Additional file 2: Table S3). Taken together, these results strongly suggest that the final ciliopathy phenotype is primarily allele-specific and the stochastic effect seems to play a more important role in the phenotypic variability of ciliopathies than the effect of modifier alleles.

### Ciliopathies represent a significant disease burden

Ciliopathies are typically autosomal recessive disorders, so their incidence is expected to increase with a higher inbreeding coefficient. We have recently devised a method to calculate the minimum burden of disease using the carrier frequency of founder mutations among a large cohort of patients with Mendelian diseases who serve as controls for mutations unrelated to their underlying illness [18]. Using this approach, we show that ciliopathies are among the most common autosomal recessive diseases in Arabia, with a minimum disease burden of 0.0004207 or 1 per 2376. (Additional file 6: Table S5 lists the breakdown of this burden per ciliopathy syndrome.) Of note, this is clearly an underestimate, since it does not account for non-founder mutations, and even for founder mutations there are a few observed in this study for which no carrier frequency could be estimated due to their absence in our database.

We also exploited the highly consanguineous nature of our population to challenge some of the previously reported “disease mutations” by showing their homozygous occurrence in individuals who lack ciliopathy phenotypes. Remarkably, of the “disease-causing” variants listed in HGMD for known ciliopathy genes, 17 were found to have a MAF of >0.01, rendering them common variants, and even for those with a MAF of <0.01 we have encountered 8 that were present at least once in the homozygous state in individuals with no ciliopathy phenotype (see Additional file 7: Table S6). As a control, we repeated the analysis using loss-of-function variants (frameshift indels, stopgains, and +1/2 splicing) and found that none of them achieved a MAF of >0.01 or was observed in the homozygous state in non-ciliopathy patients.

## Discussion

Interest in ciliopathies has increased dramatically concomitant with the increased appreciation of the role of cilia in development and homeostasis. Previous large-scale studies on ciliopathies have focused on the characterization of the ciliary proteome, knockdown cellular screens for ciliary genes, and functional genomics in the zebrafish and mouse [19–23]. This is the largest comprehensive phenomic and genomic study of ciliopathies in humans. The depth of phenotypic analysis we provide in terms of the phenotypic expression of each ciliopathy complements previous studies [24–27]. The marked phenotypic heterogeneity of ciliopathies has been the subject of interest among human geneticists. One view is that modifier alleles account for most of the variability [28]. In this model, the presence of additional ciliopathy alleles increased the severity of disease, and in extreme situations, non-Mendelian inheritance patterns were necessary for its very occurrence. Another model holds that ciliopathies are similar to other Mendelian developmental disorders and are subject to stochastic events, so that putative modifier alleles need not be limited to ciliopathy phenotypes. The unique structure of our population, where the overwhelming majority of pathogenic alleles were observed in the homozygous state, allowed us to test the contributions of each model to ciliopathy phenotypes. Our data support a more significant role for stochastic effect than previously thought. For example, interfamilial variation in the phenotype of unrelated ciliopathy patients who shared the same founder mutation was observed in only 8% (<3% when patients in our cohort were compared to previously reported patients with similar mutations). This is only slightly higher than the 3% intrafamilial variability observed, which suggests that modifier alleles appear to only play a small role and that the stochastic effect in these disorders needs to be taken into account. Regardless of the small trend toward increased variability in comparisons between families as opposed to within families, the extremely high concordance in the two groups strongly suggests that the final phenotype is primarily determined by the disease allele. It is interesting to highlight that in every instance of interfamilial as well as intrafamilial variation, the phenotypes were always caused by a splice site mutation. Thus, the discrepancy in the phenotypes may be attributed to variability in the replacement of the wild type by the mutant transcript, which may vary even among different tissue types within the same embryo during development. For example, tissue-specific differences in splicing may contribute to genotype-phenotype relationships for the common *CEP290*:c.2991 + 1665A > G allele in non-syndromic retinal disease, due to the very high levels of splicing diversity in the human retina [29, 30].

As with many disorders with genetic and allelic heterogeneity, annotating the morbid genome of ciliopathies has been challenging. Our data suggest that at least 10% of the likely causal alleles are cryptogenic and likely to evade detection by routine clinical sequencing, including exome sequencing as shown in several cases. In an outbred population, where these cryptogenic mutations likely exist in compound heterozygosity with more readily identifiable alleles, there is a tendency to invoke less likely mechanisms such as oligogenicity. However, our data are highly consistent with a fully penetrant autosomal recessive model, at least for the overwhelming majority of ciliopathies. Another challenge, again not limited to ciliopathies, is defining which allele is pathogenic. Our use of a highly inbred control cohort allowed us to challenge some of previously reported “disease mutations” in ciliopathies by demonstrating their presence in the homozygous state in ciliopathy-free individuals. Thus, one should exercise caution in interpreting HGMD-listed “disease mutations,” especially the class of missense. Again, we suspect the large number of apparently non-pathogenic ciliopathy alleles that are listed in HGMD may contribute to erroneous interpretation of their co-occurrence with pathogenic alleles as evidence of atypical inheritance, which can complicate genetic counseling unnecessarily.

The ability to identify novel disease genes is an established benefit of whole exome sequencing (WES) [31]. The majority (75%) of the novel candidate genes we report in this study (*TXNDC15, EXOC3L2, FAM98C, C17orf61*, *LRRCC1*, and *NEK4*) harbor homozygous loss-of-function variants. Additional lines of evidence include the presence of independent mutations and independent identification as ciliary genes using large knockdown screens (*TXNDC15*). Although *TXNDC15* is the only novel gene with independent mutations in this study, additional lines of evidence support the candidacy of the other novel genes. For example, TRAPPC3 (part of the transport protein particle (TRAPP) II complex, and a novel candidate gene for BBS in this study) has been shown to be required for ciliogenesis in retinal pigment epithelial (RPE) cells [32]. Furthermore, the TRAP II complex has also been shown to bind Rabin8 and target it to the centrosome as a prerequisite step for ciliogenesis [33]. This is particularly relevant to the BBS phenotype observed, since Rabin8 is known to associate with BBSome, and its knockdown in zebrafish recapitulates the BBS phenotypic readouts in this model system [33, 34]. Similarly, NEK4 interacts with the known ciliopathy protein RPGRIP1L (mutations in *RPGRIP1L* are causative for Joubert and Meckel-Gruber syndromes), is localized to the basal body in ciliated cells, and its deficiency impairs cilium assembly [35]. *CELSR2* is another proposed candidate that has been shown to be required for ciliogenesis [36]. Finally, *LRRCC1*, a novel candidate we identified in a family with Joubert syndrome, is also known as *CLERC* (centrosomal leucine-rich repeat and coiled-coil containing protein) because of its established role as a centrosomal protein in mitosis spindle organization [37]. Future studies will be required to validate the candidacy of these genes by investigating their mutation spectrum in ciliopathy phenotypes. If proven to be bona fide ciliopathy genes, the percentage of molecularly diagnosed ciliopathy patients in our cohort will increase to 88%. This is a significant improvement in diagnostic yield for a disease group that we show to be extremely common in our population, but is also relatively common in other populations.

## Conclusions

In conclusion, we studied a large cohort that spans the spectrum of ciliopathy phenotypes and identified likely causal mutations in the majority. The enrichment of our cohort for the homozygous occurrence of these alleles allowed us to conduct a robust analysis of their phenotypic expression and the extent to which this is influenced by modifiers. Our results show that the final phenotype is primarily driven by discrete single gene mutations. Our study adds many such mutations to the morbid genome of these disorders, which we show to be associated with a very high disease burden in our society.

## Methods

### Human subjects

We enrolled all patients referred to us (September 2008–April 2016) with phenotypes consistent or overlapping with known ciliopathy syndromes. The diagnostic criteria we used to group patients under specific ciliopathy diagnoses are presented in Additional file 8: Table S1. Each patient was phenotyped using a checklist that covers previously reported ciliopathy features (see Additional file 4: Table S2). We excluded cases that were referred with a known mutation to avoid inflating the yield of the genomic characterization of our cohort. Although isolated retinal dystrophy is a recognized ciliopathy phenotype, we specifically excluded these patients unless there were siblings with other ciliopathy features because retinal dystrophy differs from classical ciliopathies in being also caused by mutations in non-ciliopathy genes. Pedigrees were drawn for all enrollees, clinical photographs were obtained when possible, and blood was taken from index, parents, and available siblings and relevant relatives whenever possible. Skin biopsies were obtained for fibroblast culture in select cases. The study was approved by the King Faisal Specialist Hospital and Research Center (KFSHRC) IRB (RAC 2070023, 2080006, and 2121053) and the South Yorkshire Local Research Ethics Committee (REC reference 11/H1310/1), and informed consent was obtained from all participants prior to enrollment.

### Mutation analysis

All patients and available relatives were genotyped using the Axiom single nucleotide polymorphism (SNP) chip platform to determine the candidate autozygome as described previously [38, 39]. In parallel, the previously described “Mendeliome assay” was applied to search for likely causal variants in previously reported ciliopathy genes [40]. Briefly, we used highly multiplexed gene panels covering known ciliopathy disorders as annotated by the Online Mendelian Inheritance in Man (OMIM) catalog. The primers were designed using Ion AmpliSeq Designer software (Life Technologies, Carlsbad, CA, USA), synthesized and pooled into two multiplex reactions based upon PCR compatibility minimizing likelihood of primer-primer interactions. The libraries were run on an Ion Proton instrument (Thermo Fisher, Carlsbad, CA, USA). When negative, whole exome sequencing was performed and variants were filtered essentially as described previously [40]. When negative, autozygome-guided RT-PCR of known ciliopathy genes was attempted whenever an RNA source was available to search for cryptogenic mutations. In some cases, the causal variant was directly targeted when a founder haplotype was identified at the genotyping stage. Variants in known ciliopathy genes were considered likely causal if they were nonsense, frameshift, or canonical ± 1 or 2 splice sites, absent as homozygous in the ExAC and Saudi genome databases, and having a minor allele frequency (MAF) <0.01 in the heterozygous state. For missense or in-frameshift variants, they had to be absent as homozygous in the ExAC and Saudi genome databases and have a MAF <0.01 in the heterozygous state, predicted to be pathogenic and deleterious by the sorting intolerant from tolerant (SIFT) and Polyphen tools, and with a Combined Annotation Dependent Depletion (CADD) score >15. For non-canonical splice site variants, they had to be absent as homozygous in the ExAC and Saudi genome databases, have a MAF <0.01 in the heterozygous state, and either reported in the Human Gene Mutation Database (HGMD) with confirmed abnormal splicing effect on reverse transcriptase PCR (RT-PCR), or demonstrated by us on RT-PCR to result as a minimum in (1) an aberrant band exclusive to the patient and confirmed by sequencing and/or (2) complete absence of the normal transcript in the patient despite the proper use of internal controls.

### Cellular characterization of ciliary genes

A standard ciliogenesis assay on patient-derived fibroblasts was performed as described previously [9]. To assess the involvement of TXNDC15 in cilia formation, we stained control and mutant fibroblasts for immunofluorescent microscopy of the cilia marker ARL13B. We modeled transcript loss by using small interfering (siRNA) reagents against the human *TXNDC15* transcript (ON-TARGETplus SmartPool, GE Healthcare: 5’-AGAGGAAAGUGGUCGCUUA-3’, 5’-CGACAGAGGACUCCAAUAA-3’, 5’-CGGUAGUGACUGUACUCUA-3’, and 5’-CCAGAAUUGGUUAGUGUGA-3’). We transfected the immortalized ciliated cell line hTERT-RPE1 (retinal pigment epithelial), following previously described protocols [41], and after 72 h incubation used qRT-PCR to test abundance of *TXNDC15* mRNA. “Gateway” cloning (Thermo Fisher Scientific) was used to insert a TXNDC15 “Gateway” entry vector (NM_024715.3, obtained from GeneCopoeia Inc., Rockville, MD, USA) into a destination construct that had C-terminal strep-II/FLAG tags for tandem affinity purification (CTAP). Human hTERT-RPE1 cells were transfected with the TXNDC15-CTAP expression construct, as described previously [41], and stained for the FLAG tag. To identify protein interactants of TXNDC15, the cells were lysed, and streptavidin- and FLAG-based tandem affinity purification steps were performed as described previously [42]. Liquid chromatography-MS/MS analysis was performed using an UltiMate 3000 Nano HPLC system and a mass spectrometer (LTQ Orbitrap XL, Thermo Fisher Scientific). The results from three biological replicates were filtered on the basis of *n* ≥ 2 peptides in at least two replicates and absence in a negative control dataset (pull-downs of RAF1-CTAP and untransfected cells; Additional file 2: Table S3).

## Additional files

Additional file 1: Figure S1. Bar graph showing the percentage of the main features for each distinct ciliopathy syndrome. (PPTX 70 kb)
Additional file 2: Table S3. Clinical and genomic data for all cases in the study. (XLSX 83 kb)
Additional file 3: Supplemental clinical data: clinical details for the affected cases with mutations in novel candidate genes. (DOCX 35 kb)
Additional file 4: Table S2. Checklist used to cover all previously reported ciliopathy features. (DOCX 36 kb)
Additional file 5: Table S4. Identification of TXNDC15 interacting proteins using tandem affinity purification (*TAP*). The list of 224 unique proteins is significantly enriched in known or predicted ciliary proteins. (XLSX 30 kb)
Additional file 6: Table S5. Ciliopathy disease burden in the population for each pathogenic variant identified in known ciliopathy genes. (XLSX 19 kb)
Additional file 7: Table S6. List of common variants with MAF of >0.01 that are listed as “disease-causing” variants in HGMD for known ciliopathy genes. (XLSX 12 kb)
Additional file 8: Table S1. List of diagnostic criteria used to clinically classify each ciliopathy. (XLSX 10 kb)
